# The deregulation of arachidonic acid metabolism in ovarian cancer

**DOI:** 10.3389/fonc.2024.1381894

**Published:** 2024-05-02

**Authors:** Qiuyi Xia, Wen Gao, Jintao Yang, Zhifang Xing, Zhaodong Ji

**Affiliations:** ^1^ Department of Laboratory Medicine, Huashan Hospital, Fudan University, Shanghai, China; ^2^ Cancer Hospital of the University of Chinese Academy of Sciences (Zhejiang Cancer Hospital), Institute of Basic Medicine and Cancer (IBMC), Chinese Academy of Sciences, Hangzhou, Zhejiang, China; ^3^ Key Laboratory of Digital Technology in Medical Diagnostics of Zhejiang Province, Hangzhou, Zhejiang, China

**Keywords:** arachidonic acid, ovarian cancer, metabolic pathway, biological marker, targeted therapy

## Abstract

Arachidonic acid (AA) is a crucial polyunsaturated fatty acid in the human body, metabolized through the pathways of COX, LOX, and cytochrome P450 oxidase to generate various metabolites. Recent studies have indicated that AA and its metabolites play significant regulatory roles in the onset and progression of ovarian cancer. This article examines the recent research advancements on the correlation between AA metabolites and ovarian cancer, both domestically and internationally, suggesting their potential use as biological markers for early diagnosis, targeted therapy, and prognosis monitoring.

## 1 Lipid metabolites in ovarian cancer

Ovarian cancer(OC) is a common gynecological malignant tumor with a hidden onset, high degree of malignancy, and high mortality rate (1). Globally, there are nearly 240,000 new cases annually, with the ovarian cancer mortality rate expected to significantly increase by 2040 (2). However, the lack of specific markers for ovarian cancer means that most patients are diagnosed in the late stages of the disease (51% in stage III or 29% in stage IV), missing the optimal timing for surgery, resulting in a 5-year survival rate of only 26-42% (3). Therefore, there is an urgent need to develop new molecular markers for the prevention and treatment of ovarian cancer. Extensive research indicates a close relationship between abnormal lipid metabolism and tumor development, increasing the possibility of detecting subtle metabolic changes in early tumor formation, making it a promising candidate as an ideal biomarker for ovarian cancer diagnosis (4–6). Among them, arachidonic acid is considered a key lipid metabolism product, playing a significant role in cell proliferation, apoptosis, invasion, and metastasis. Therefore, research on arachidonic acid and other lipid metabolites can not only help us better understand the pathogenesis of ovarian cancer but also offer new insights and methods for the treatment and prevention of this disease.

## 2 Arachidonic acid metabolism in cancer

**Figure 1:**
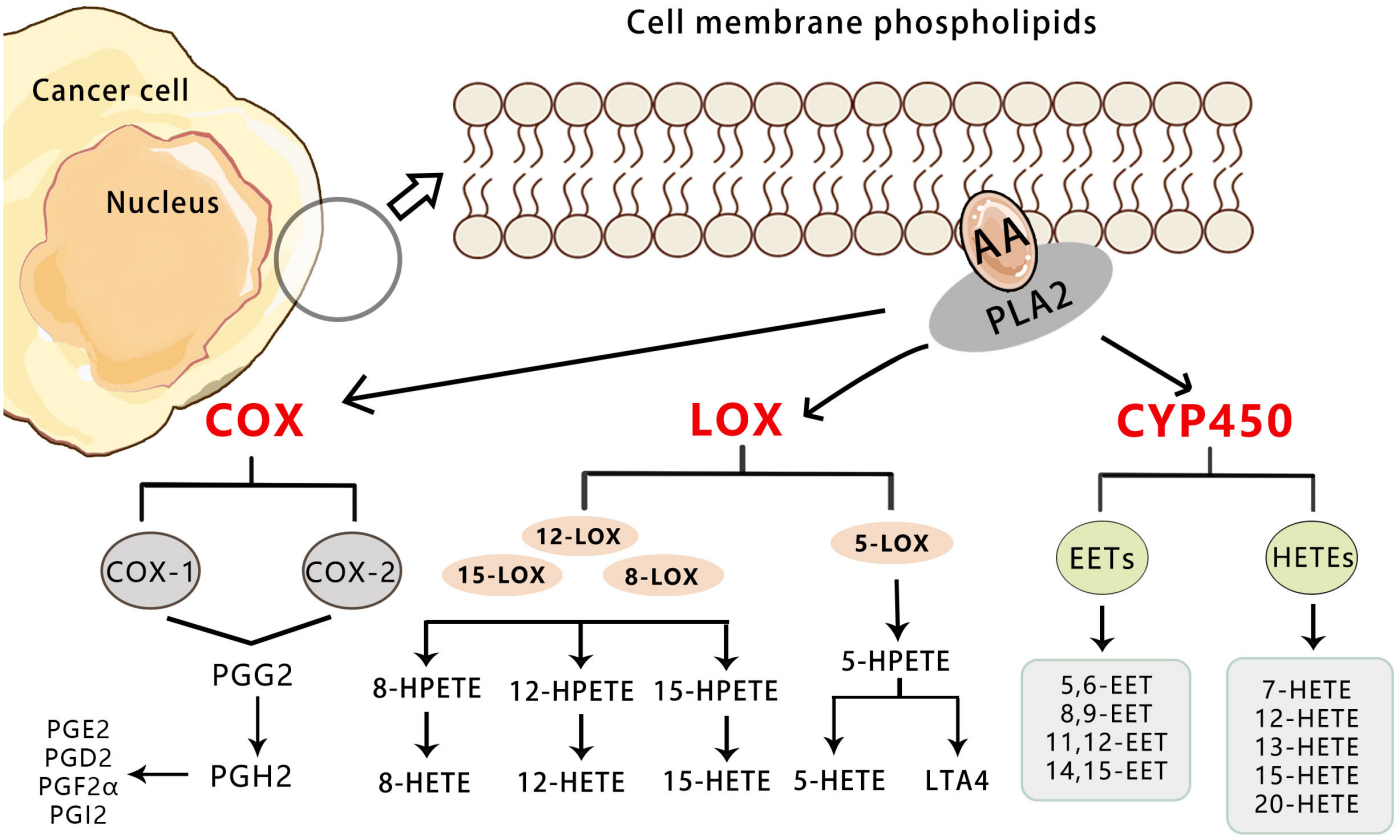
Overview of the three pathways for AA metabolism in cancer. Upon cell stimulation, phospholipase A2 induces FFAs, primarily AA derived from the lipid bilayer. The released AA is metabolized into bioactive lipid signaling molecules by three different enzymatic pathways. COX-1/2 metabolize AA to a series of prostaglandins (PGD2, PGE2, PGF2, PGH2, and PGI2).5-LO metabolizes AA to 5-HETE, LTB4, and cysteine leukotrienes, and 12-LO metabolizes AA to 12-HETE. Both subtypes of 15-LO metabolize AA to 15-HETE and, to a lesser extent, 12-HETE. Cytochrome P450 metabolizes AA to 19-/20-HETEand so on.

Arachidonic acid (AA) is an essential omega-6 polyunsaturated fatty acid in the human body (7). The endogenous generation of arachidonic acid (AA) is derived from phospholipids in the cell membrane, which is catalyzed by the superfamily of phospholipase A2 (PLA2). This process is induced by various cellular activation signals, including stimulation of tumor necrosis factor receptor (TNFR) and toll-like receptor 4 (TLR4) in the course of inflammation or infection. Among the members of the PLA2 enzyme superfamily, three contribute to eicosanoid production and are involved in distinct functions within eicosanoid metabolism (8–10). The cytosolic calcium-dependent PLA2 alpha (cPLA2α) primarily facilitates the production of free fatty acids (FFAs) and generation of AA, which plays a crucial role in cellular signaling (11). The cytosolic calcium-independent PLA2 alpha (iPLA2α) contributes to cellular homeostasis through synthesis of specialized pro-resolving mediators (SPMs) and reacylation of free AA, while secretory PLA2 (sPLA2), operating in a paracrine manner, controls the release of free AA and induces local inflammatory responses. In addition to PLA2 enzymes, other enzymes known as phospholipase C (PLC) and phospholipase D (PLD), generate AA via intermediate products such as diacylglycerol(DAG) (12–14). Free AA is converted in three ways: ① Cyclooxygenase (COX) enzymes catalyze the metabolic conversion of arachidonic acid to prostanoids including prostaglandins (PGs), prostacyclin, and thromboxane(TXs). ② Lipoxygenase (LOX) pathways catalyze the conversion of arachidonic acid to leukotrienes and lipoxins. ③ Cytochrome P450 enzyme (CYP) pathway (15) (**Figure 1**). Previous studies have demonstratedthat AA and its metabolites promote the occurrence and development of tumors by regulating the process of cell carcinogenesis, progression, and differentiation such as cell proliferation, chemotaxis, mitosis, migration, and apoptosis (16–18). Therefore, AA metabolism is considered to be one of the active metabolisms in tumor metabolism.

### 2.1 The action of COX pathway in tumor

**Figure 2:**
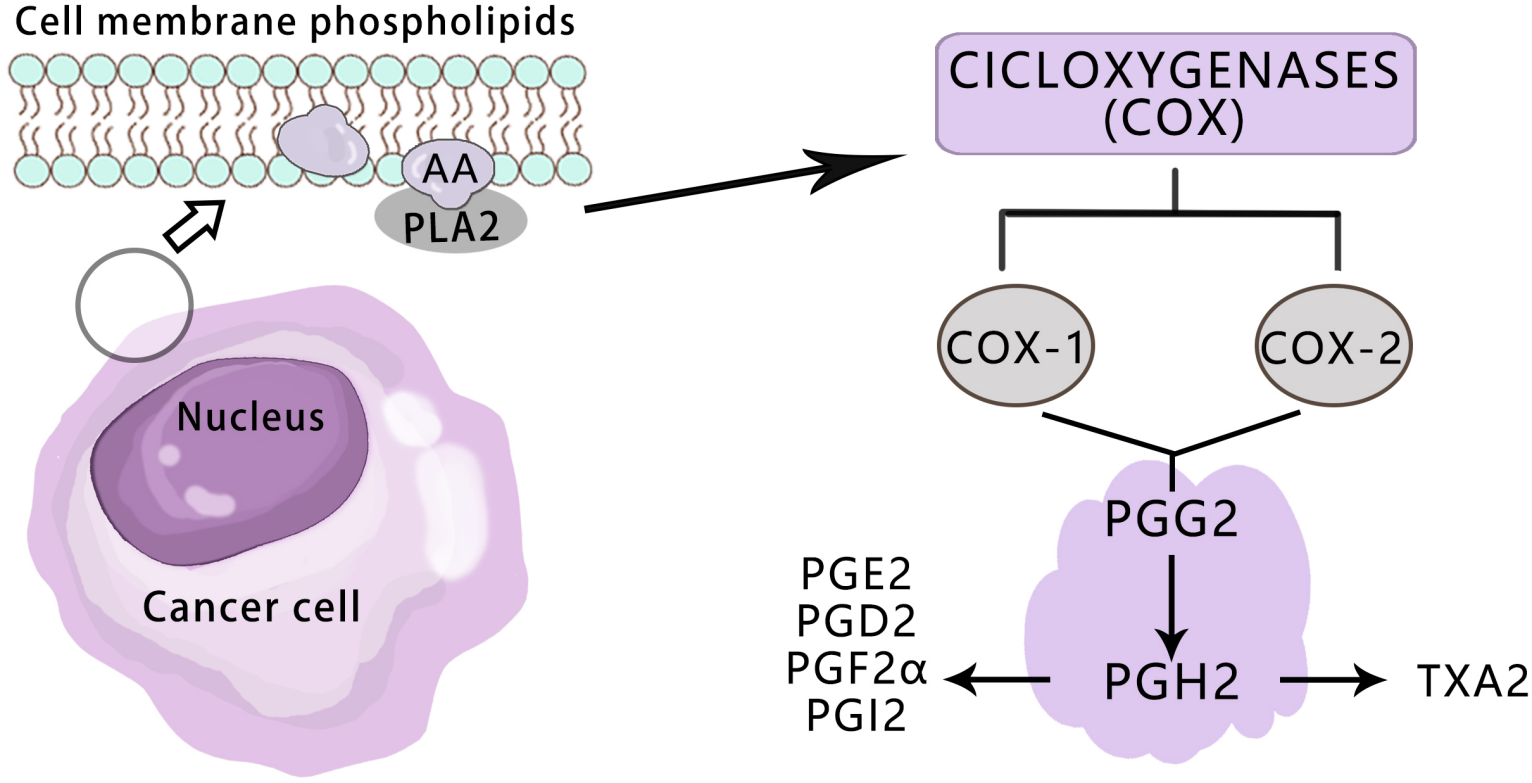
Overview of the pathways for the COX-mediated AA metabolism in cancer.

The COX enzyme is the initial catalyst in the arachidonic acid pathway for prostaglandin (PG) and thromboxane (Tx) synthesis, existing in three isomeric forms: COX-1, COX-2, and COX-3 (19). Both COX-1 and COX-2 enzymes facilitate the conversion of cell membrane phospholipids to arachidonic acid via phospholipase A2, followed by its conversion to PGH2 through PGG2 (20)(**Figure 2**).COX-1 is a constitutive enzyme essential for normal physiological functions and widely distributed across various tissues to safeguard cellular integrity (21). There is no significant difference in the expression level of COX-1 between tumor tissues and normal tissues (22).

COX-2 is an inducible enzyme that performs a crucial function in the pathophysiological process of various cancers, including pancreatic, breast, prostate, lung, liver, cervical, bowel, and skin cancer (23–28). It is either not expressed or expressed at low levels in most normal tissues. The expression of COX-2 is associated with inflammation, cell survival, proliferation, angiogenesis, invasion, and metastasis. In response to growth factors and endotoxins, COX-2 is briefly but strongly expressed. The overexpression of COX-2 significantly enhances the production of PGE2, leading to increased cell aggressiveness (29). PGE2 contributes to the development and progression of many cancers by activating the membrane receptors EP (including EP1, EP2, EP3, and EP4 receptors) and the nuclear receptor PPARδ of target cells (30). Other studies have indicated that high expression of COX-2 can hinder the apoptosis of tumor cells, which is crucial in the early stages of tumor formation (31). The overexpression of COX-2 can result in increased synthesis of PG, which plays a significant biological role in tumor growth and proliferation. PGE2 can impede apoptosis induced by selective COX-2 inhibitors by upregulating the anti-apoptotic protein bc-l2 (32). Kajita et al. discovered that the level of specific enzymes in the PG synthesis pathway, such as TXA2 synthetase, increased in papillary thyroid carcinoma. Additionally, the expression of COX-2 protein in papillary thyroid carcinoma was higher than in normal thyroid tissue and varied greatly, indicating that COX-2 could promote the growth of the thyroid papillary gland (33). These findings suggest that the increased expression of these enzymes may contribute to the pathogenesis of tumors.

What’s more, angiogenesis is the physiological basis of solid cancer growth and metastasis (34). The high expression of COX-2 and its metabolite PGE2 promotes angiogenesis by up-regulating angiogenic factors such as vascular endothelial growth factor (VEGF) and basic fibroblast growth factor (bFGF) (35). COX-2 also promotes the metastasis and invasion of ovarian cancer by inducing matrix metalloproteinases (MMPs) in the extracellular matrix and the decomposition of collagen matrix, which may be involved in the activation of the PI3K/AKT signaling pathway (36–38). Inhibition of COX-2 with its specific inhibitor NS-398 can increase the expression of E-cadherin and inhibit the expression of slug, vimentin, MMP2, and MMP9, thereby suppressing the invasion and metastasis of ovarian cancer cells under estrogen treatment (39–41). Moreover, overexpression of COX-2 in ovarian cancer cells can directly up-regulate Bcl-2 expression through the increased synthesis of PGs. In addition, COX-2 can inhibit the proliferation of T and B lymphocytes through its product PGE2, and also inhibit the synthesis of cytokines to reduce the cytotoxicity of natural killer cells (42). Therefore, the role of COX-2 and PGE2 in promoting tumor proliferation and invasion, reducing tumor cell apoptosis, and promoting tumor angiogenesis has been confirmed, and it proved that they play an important role in carcinogenesis, and have the function of promoting tumor by inhibiting the body’s immunity (43).

### 2.2 The action of LOX pathway in tumor

LOX is the initial enzyme in the leukotriene (LT) pathway of arachidonic acid. Isoenzymes of LOX consist of 5-LOX, 12-LOX, and two isomers of 15-LOX (15-LOX-1, 15-LOX-2). However, 5-LOX, 12-LOX, and 15-LOX-1 have pro-tumor effects, while 15-LOX-2 appears to have anticancer effects (44).

**Figure 3:**
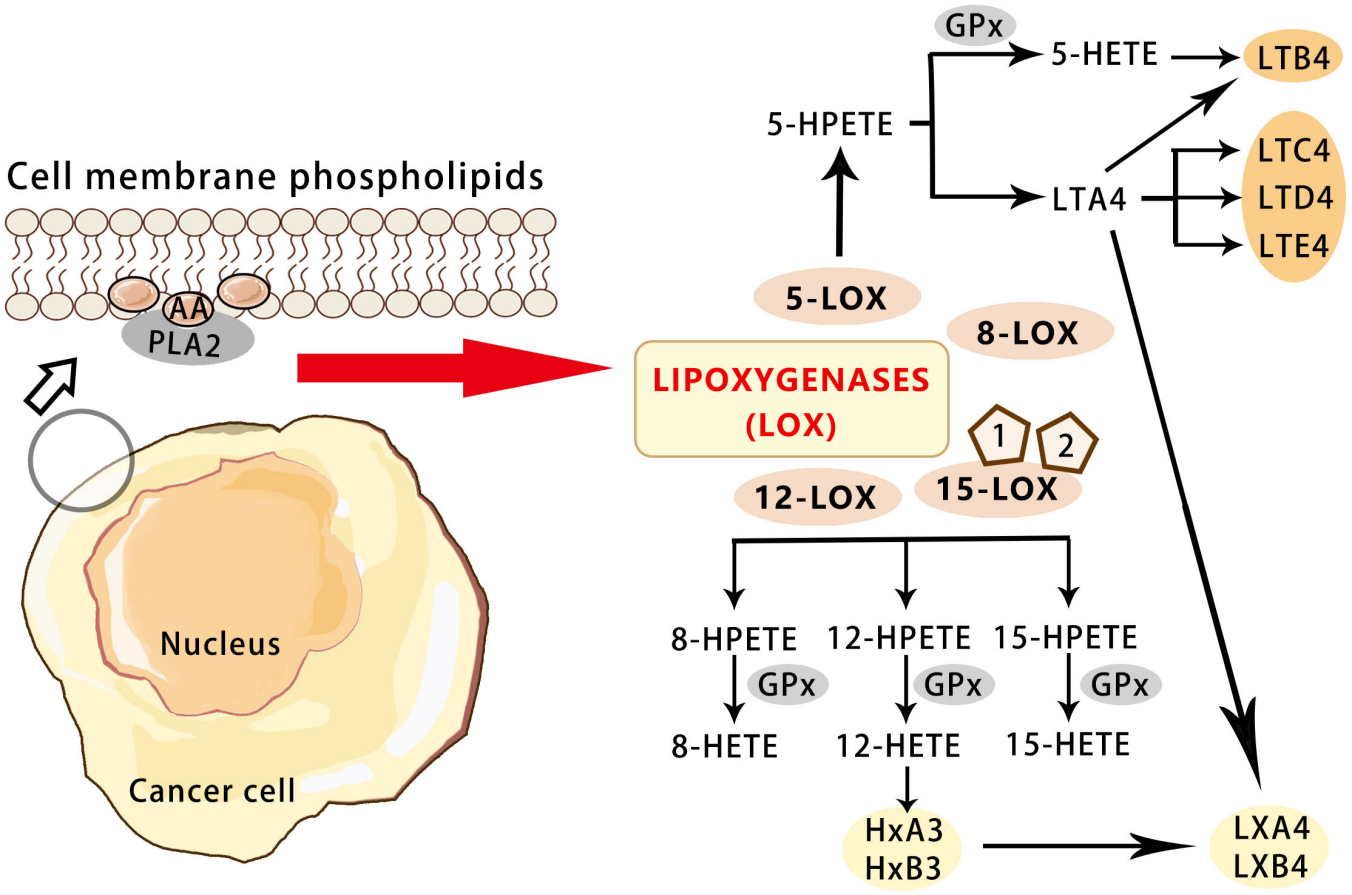
Overview of the pathways for the LOX-mediated AA metabolism in cancer.

LOX catalyzes the oxidation of arachidonic acid to 5-HPETE, which is subsequently metabolized to 5-HETE (5-hydroxyeicosatetraenoic acid) and LTB4 (leukotriene B4) (45). As a member of the arachidonic acid oxygenase family, 5-LOX is composed of 674 amino acids and is a monomeric enzyme containing iron ions. 5-LOX can be transcriptionally regulated by transcription factor Er, Sp1, nuclear factor-κB (NF-κB), and GATA (46). 5-LOX is activated by 5-LOX activating protein (FLAP) to catalyze arachidonic acid, which is released from the phospholipid bilayer by phospholipase A2 (47). Arachidonic acid is transformed into 5-HPETE, which can be metabolized by glutathione peroxidase into 5-HETE (48). The activity of 5-LOX leads to the formation of unstable LTA4, which can be converted to LTB4, LTC4,LTD4 and LTE4 (49). More significantly, the average endogenous level of LOX metabolites such as 12-HETE (12-hydroxyeicosatetraenoic acid) in primary prostate cancer were found to be significantly higher than that in non-neoplastic prostate tissue. It is suggested that 12-HETE is crucial in the progression of prostate cancer and the LOX pathway may be a target for the treatment and prevention of prostate cancer (50). 12-HETE has also been shown to play an important role in cancer adhesion, invasion, and metastasis. It stimulates NF-κB activation and NF-κB-dependent ICAM-1 expression through RhoA and PKCα signaling pathways, and the Rho/Rac family of GTases also play a role in cell adhesion and migration. 12-HETE can also promote the secretion of protease and enhance the motor capacity of tumor cells (51)(**Figure 3**).

Various environmental and chemical carcinogens activate pro-tumor mediators during carcinogenesis, including 5-LOX, whose metabolite 5S-HERE acts as a substrate for COX-2 to form bicyclic and is further transformed into two pro-angiogenic mediators (52). However, the specific role of these products in cancer development needs better understanding, given the pro-angiogenic role of these enzymes. As a result, 5-LOX is considered a new target for cancer prevention and treatment, while LETA4 hydrolase is considered a tumor promoter whose inhibitors can reduce tumor growth and development (53). Although most of the metabolites synthesized by the COX and LOX pathways can promote tumorigenesis. Additionally, CYP450 monooxygenase-derived metabolites can also regulate the occurrence and growth of cancer, playing both a pro-tumor and anti-tumor role (54).

### 2.3 The action of CYP pathway in tumor

**Figure 4:**
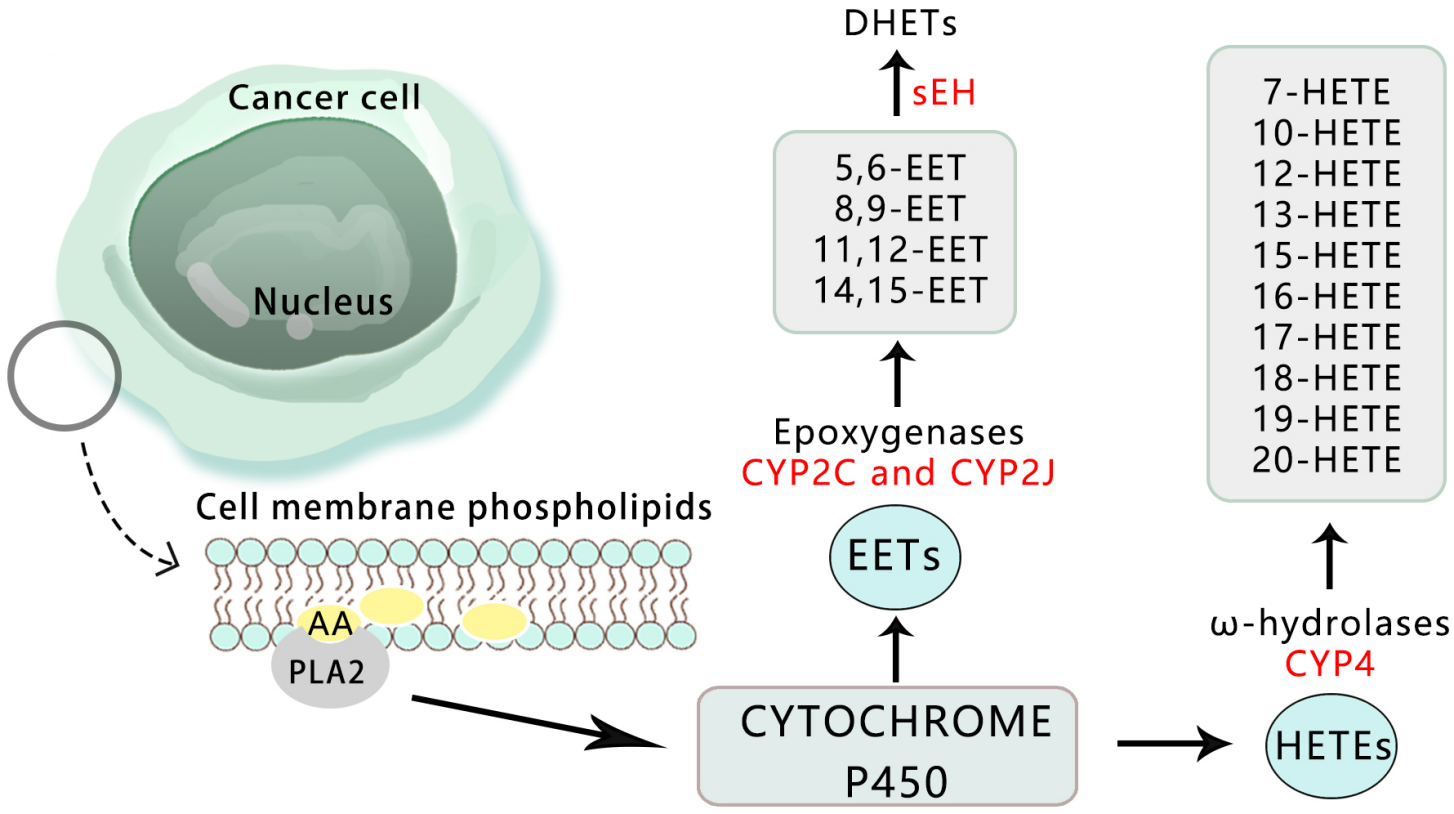
Overview of the pathways for the CYP450-mediated AA metabolism in cancer.

Cytochrome P450 (CYP) belongs to the group of hemoglobin enzymes. It is a super-family gene encoding isoenzymes with a structure and function related to CYP. The combination of reduced CYP and carbon monoxide (CO) has a special light absorption peak at 450 nm (55). The expression of CYP surface oxidase is significantly high in cancer tissues, while almost no expression is found in adjacent normal tissues. This confirms that CYP surface oxidase and its metabolites can regulate the occurrence and growth of cancer, playing both a pro-tumor and anti-tumor role. It has been shown that reducing the expression and activity of CYP450 has anti-tumor effects, making it a promising anti-cancer treatment method (56) (**Figure 4**).

Arachidonic acid produces epoxyeicosatrienoic acid (EETs) and hydroxyeicosatetraenoic acid (HETEs) through the catalysis of cytochrome P450 surface oxidase. EETs are further categorized into four types: 5,6-EET, 8,9-EET, 11,12-EET, and 14,15-EET (57, 58). Recent studies have focused on the role and mechanism of EETs in important biological processes of tumors, including proliferation, apoptosis, and metastasis (59). These studies have revealed that EETs can significantly enhance the proliferation of tumor cells and protect them from TNF-α-induced apoptosis by up-regulating anti-apoptotic proteins Bcl-2 and Bcl-XL, while down-regulating pro-apoptotic proteins Bax and Bak, thereby reducing the activity of caspase-3 protein (60).

In light of the characteristics of the CYP gene superfamily, research on the correlation between CYP and gynecologic tumors primarily focuses on specific CYP polymorphisms involved in sterol hormone metabolism. The polymorphisms of CYPlAl, CYPlBl, CYPl7, and CYPl9 have garnered increasing attention in relation to gynecologic tumors (61, 62). CYPlAl is an enzyme involved in the metabolism of polycyclic aromatic hydrocarbons (PAHs), which are significant metabolically active chemical carcinogens, and is expressed in various sterol hormone-reactive tissues such as the ovary, mammary gland, and prostate. Studies have shown that the CYPlAl Ile/Val and Val/Val genotypes are significantly more common in patients with epithelial ovarian tumors, indicating that women with these genotypes are at higher risk for ovarian cancer (63). CYP and its lipid metabolites play a role in the development of ovarian tumors, and their expression may be a crucial marker for tumor development (64).

## 3 Arachidonic acid metabolism in OC

### 3.1 Cox pathway in ovarian cancer

AA catalyzes the synthesis of prostaglandins (PGs) and thromboxanes (TXs) through cyclooxygenase (COX). There are two subtypes of COX: COX-1 and COX-2. COX-1 is widely distributed in tissues, with no significant difference in expression levels between tumor tissues and normal tissues. On the other hand, COX-2 is closely associated with the development of inflammation and tumors. Overexpression of COX-2 significantly promotes the biosynthesis of prostaglandin E2 (PGE2). PGE2 stimulates the occurrence and progression of various cancers by activating membrane receptors EP and nuclear receptor PPARδ in target cells.

Recently, Mauricio A. Cuello discussed the impact of COX-2 on the immune spectrum of high-grade serous ovarian cancer (HGSOC). Elevated levels of COX-2 may hinder NK cell activity, promote the expression of cytotoxic T lymphocyte-associated protein-4 (CTLA-4), affecting the efficacy of anti-CTLA-4 immunotherapy in HGSOC patients. Targeting COX-2 before anti-CTLA-4 immunotherapy could be a promising strategy to enhance the effectiveness of immunotherapy in ovarian cancer patients (42). Additionally, Ning Ding’s research found that the overexpression of COX-2 in SKOV3 cells (human ovarian cancer cells) is associated with the acquisition of stem cell properties, inflammatory microenvironment, enhanced tumor sphere formation, increased cell proliferation, and enhanced metastatic potential, all contributing to ovarian cancer progression and metastasis (65). Based on current research, the roles of COX- and LOX-derived class of diterpenes in cancer have been fairly well studied. COX-1, COX-2, mPGES-1, EP1, and EP2 are mainly expressed in epithelial cells of human epithelial ovarian cancer (66). The expression of COX-2 in ovarian cancer cells is regulated by various cytokines, such as EGF, vitamin D, IL-1β, which can stimulate the proliferation, migration, and angiogenesis of ovarian cancer cells. It mainly enhances the proliferation and migration of human ovarian cancer CAOV-3 cells by activating the phosphatidylinositol 3 kinase/protein kinase B (PI3-k/Akt) pathway (67). Moreover, by analyzing epithelial ovarian cancer tissues and cell lines, COX-2 also regulates cell growth and apoptosis through the PI3K/AKT signaling pathway in ovarian cancer tissues (68).

### 3.2 LOX pathway family in ovarian cancer

In the Lipoxygenase (LOX) pathway, AA is catalyzed by the LOX enzyme, undergoing an oxidation reaction at specific sites to form hydroxyl-containing products, namely Hydroxyeicosatetraenoic acid (HETEs, including 5-, 8-, 12-HETE, etc.). Additionally, there are lipoxins (LXs), leukotrienes (LTs), and other products, which will not be the focus here.

Research in ovarian cancer has found an increased expression level of 5-LOX in the immune stroma of tissues, suggesting a specific impact on the tumor microenvironment during tumor initiation and progression (47). Under hypoxic conditions, the transcription levels of 5-LOX and ALOX5AP in ovarian cancer cell lines also increase, significantly correlating with poor overall survival, progression-free survival, lymphatic infiltration after initial treatment, rapid relapse, and other adverse clinical pathological features. Current studies have discovered that various LOX pathway metabolites, such as HETEs, play a role in inhibiting tumor cell apoptosis, stimulating angiogenesis, enhancing cell proliferation, and promoting metastasis. The presence of 12-HETE has been identified in high-grade serous ovarian cancer and normal ovarian epithelial tissues. The 12-LOX - 12-HETE system is present in two epithelial ovarian cancer cell lines, OVCAR-3 and SK-OV-3, with high levels of 12-LOX mRNA and protein (69, 70). Exposure to arachidonic acid increases the production of 12-HETE in both epithelial ovarian cancer cell lines. Numerous studies have confirmed its ability to promote the proliferation, adhesion, migration, and angiogenesis of various cancer cells such as breast, prostate, and colon cancer. These findings collectively indicate that metabolites produced by the LOX pathway can play a role in the development of ovarian cancer through different isoenzymes (71).

### 3.3 CYP450 pathway in ovarian cancer

Most studies on gynecologic malignant tumors have focused on the arachidonic acid oxygenase pathway, particularly examining the roles of PGE2 and PGF2a along with their respective receptors. While some research has touched upon the P450 cytochrome epoxygenase pathway in ovarian cancer, there is a lack of in-depth mechanistic studies in this area (72). Research indicates that CYP2C8 and CYP3A5 are prominently expressed in the majority of ovarian tumors, showing higher IHC staining intensity of CYP3A5 and other CYP enzymes in primary ovarian cancer tissues. In contrast, CYP3A4 is expressed at significantly lower levels in ovarian cancer (73, 74).

Arachidonic acid is converted into four eicosanoids (EETs) by cytochrome P450 oxygenases (CYP oxygenases). Human EETs are synthesized by the CYP2 family, including the CYP2C and CYP2J families. EETs are intermediate products downstream of the VEGF signaling pathway, regulating vascular tone and maintaining vascular homeostasis. They have been shown to promote endothelial cell proliferation, enhance endothelial cell migration through pathways such as eNOS, MEK/MAPK, and PI3K, and boost VEGF-mediated angiogenesis. Angiogenesis plays a crucial role in tumor development, enabling tumors to access more nutrients and oxygen for continued growth, spread, and metastasis by providing pathways for tumor cells to travel through the bloodstream to distant sites.

## 4 The arachidonic acid metabolism in tumor microenvironment of ovarian cancer

AA is a highly concentrated polyunsaturated fatty acid present in the microenvironment of ovarian cancer, with multiple research findings indicating its association with adverse clinical outcomes (75). Enzymes and metabolites related to the AA metabolic pathway regulate various pathophysiological processes in the cellular system, establishing the tumor microenvironment (TME) (76). The progression, metastasis, and spread of cancer, as well as the evasion of tumor cells from immune surveillance, crucially depend on the signaling network of the tumor microenvironment. In ovarian cancer, ascites is a significant component of the peritoneal TME, containing numerous tumor spheroids and immune cells, particularly tumor-associated macrophages (TAMs), T cells, and NK cells (77). Studies have reported a correlation between ovarian cancer survival and the abundance of immunosuppressive CD163-CD206-high tumor-associated macrophages (TAMs) and high levels of arachidonic acid (AA) in the tumor microenvironment. A research also indicates that the high expression of CD163 and CD206/MRC1 in TAMs is closely related to the inhibition of cytokine-triggered signals, reflecting impaired interferon and IL-6 transcriptional responses in monocyte-derived macrophages by AA (78). How does arachidonic acid affect the cytokine-triggered signals in macrophages? It influences the signal transduction of cytokine-triggered signals in macrophages by inhibiting the transcriptional responses to interferon and IL-6. This inhibition of pro-inflammatory signals is caused by dysfunction of homologous receptors, manifested as inhibition of JAK1, JAK2, STAT1, and STAT3 phosphorylation, as well as the relocation of interferon receptor IFNAR1, STAT1, and other immune regulatory proteins in lipid rafts. Exposure to AA leads to significant accumulation of free AA in lipid rafts, which appears to be a crucial mechanism, as inhibiting its binding to phospholipids does not affect AA-mediated interference with STAT1 phosphorylation. Therefore, the association between arachidonic acid and TAMs in the ovarian cancer microenvironment involves high levels of arachidonic acid impairing the signal transduction and transcriptional responses of TAMs, leading to an immunosuppressive environment. Additionally, macrophages play a key role in tumor growth, metastasis, immune suppression, and chemoresistance, contributing significantly to the progression and treatment of drug resistance in ovarian cancer (79).

## 5 Targeted therapy and drugs for arachidonic acid metabolism

An article entitled “Targeting LTA4H facilitates the reshaping of the immune microenvironment mediated by CCL5 and sensitizes ovarian cancer to Cisplatin” discusses the establishment of a prognosis model for ovarian cancer based on pufa-related genes, the role of the downstream LTA4H gene in the progression and drug resistance of ovarian cancer, and potential treatment strategies for ovarian cancer (80). The article highlights the significant role of LTA4H in influencing tumor characteristics and the immune microenvironment in the context of ovarian cancer. Positive correlation between LTA4H and poor prognosis in ovarian cancer has been observed, with the lack of LTA4H enhancing sensitivity to Cisplatin. Knockdown of LTA4H has been shown to inhibit the proliferation of ovarian cancer cells, while high expression of LTA4H leads to a decrease in infiltrating CD8+ T cells, which are crucial for anti-tumor immune responses. Furthermore, LTA4H is associated with abnormal metabolism in the arachidonic acid (AA) pathway, resulting in a reduction of certain chemokines such as CCL5. The decrease in chemokines may lead to changes in the composition of immune cells in the tumor microenvironment. LTA4H has been identified as a potential therapeutic target. Targeting LTA4H, whether through genetic manipulation or chemical inhibition, can yield favorable therapeutic effects for ovarian cancer. However, targeted therapies and drug researches related to arachidonic acid metabolism in ovarian cancer are still in the exploratory stage and have not yet been widely applied in clinical settings. Due to the complexity of the biochemical properties of AA and its metabolites, inhibitors of AA have been continuously discovered to have effects in tumors. Additionally, COX-2, HETEs, and key enzymes in their metabolic pathways may become potential targets for early cancer detection and treatment.

COX-2, LOX-5, and HETEs, along with the key enzymes in their metabolic pathways, could be potential targets for the early detection and treatment of cancer. The standard initial treatment for ovarian cancer is a combination of platinum and taxane (81, 82). However, due to the complexity of the biochemical properties of AA and its metabolites, the role of its inhibitors in tumors is continually being uncovered.

The COX inhibitor SC-560 was initially used as a pharmacological tool to study the role of COX-1-derived prostaglandins in inflammation and pain. It was later found that at specific doses of COX-1 inhibition, SC-560 exhibited mild to moderate inhibitory effects on tumor growth. Its anti-tumor activity has been demonstrated in various ovarian and colorectal cancer *in vitro* models and other types of tumor tissue. SC-560 also enhanced the sensitivity of paclitaxel-resistant ovarian cancer cell lines with MDR1/p-glycoprotein upregulation to paclitaxel. It belongs to a group of small molecules that may target specific genes in ovarian cancer stem cells (OVCSC), suggesting that SC-560 could be a promising lead compound for ovarian cancer (83).

Reduced expression of COX-2 and PGE is positively associated with decreased severity and occurrence of ovarian cancer. Currently, COX-2 has been recognized as a new cancer chemoprevention and therapeutic target. Selective COX-2 inhibitors like celecoxib, when used in combination with anti-cancer drugs, can overcome multidrug resistance in various cancers. They can also reduce cell growth, increase cleaved caspase-3 activity, and induce cell cycle G1 phase arrest in a dose-dependent manner in ovarian cancer cells (84).

Additionally, recent studies shown that the CYP4A/F-20-HETE pathway has a positive feedback regulatory effect (85). The AA pathway inhibits tumor cell migration and invasion by inhibiting 20-HETE synthesis. This is achieved by using the selective 20-HETE inhibitor N-hydroxy-N’-(4-butyl-2-methylphenyl)formamidine (HET0016), which significantly reduces the levels of vascular endothelial growth factor responsible for tumor cell communication with the microenvironment (86, 87), whether used alone or in combination. It is worth noting that ovarian cancer shows upregulation of CYP4A/F family enzymes involved in 20-HETE production and widespread use of HET0016 as a therapeutic approach against excessive proliferation (88).

Studies have also indicated that berberine, a natural compound with low cytotoxicity in normal cells, effectively inhibits the regeneration of post-chemotherapy ovarian cancer cells, particularly SKOV3 cells induced by VP16 (82, 89). Berberine can lower AA levels while increasing PGE2 levels, thus reversing the caspase-3-iPLA2-AA-COX-2-PGE2 pathway induced by chemotherapy drugs in SKOV-3 cells (90). This confirms that berberine can prevent recurrence of ovarian cancer, suggesting that combining chemotherapy drugs with berberine may prove to be an effective approach for preventing its recurrence (89).

## 6 Functional and signaling pathway enrichment analysis of AA metabolism related genes in OC

**Figure 5:**
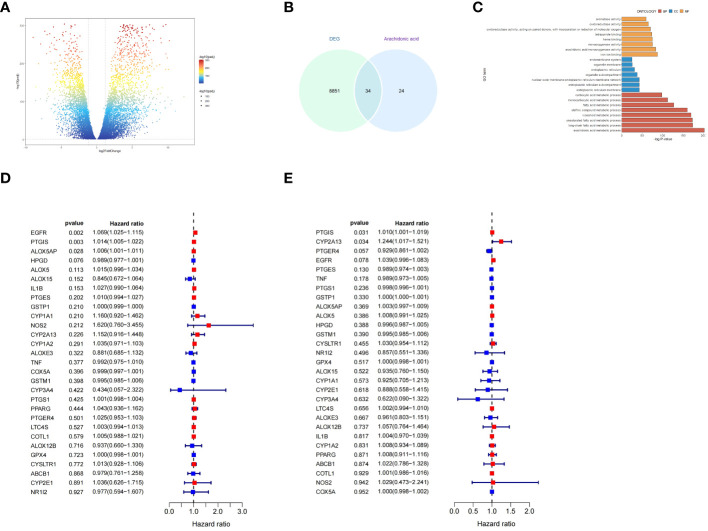
Functional and signaling pathway enrichment analysis of AA metabolism related genes in OC. **(A)** Volcano plot of DEGs between ovarian cancer and non-tumor tissues based on the TCGA (OV for ovarian cancer, accessed on 20240329) and GTEx (Ovary, accessed on 20240329) database. **(B)** Venn diagram showing the number of DEGs and AA metabolism related genes; the intersection part is the total number of genes in the two datasets. **(C)** Results of GO analysis of overlapping DEGs. **(D, E)** Forest plot demonstrating the univariate Cox model results of 34 OS/PFS-related key genes of AA metabolism.

To comprehensively evaluate the function and signaling pathways of AA metabolism related genes in OC, we obtained transcriptome expression profile data from TCGA-OV cohort and normal ovarian tissues, and after analysis, a total of 8885 differentially expressed genes (DEGs) were identified (**Figure 5A**).Then, veen analysis was utilized to identify the above DEGs and AA metabolism related genes,resulting in a total of 34 overlapping DEGs (**Figure 5B**). To further determine the function of 34 candidate genes with related signaling pathways in OC, enrichment analysis was performed. Gene Ontology (GO)-biological process (BP) analysis showed that candidate genes were primarily involved in carboxylic acid metabolic process and monocarboxylic acid biosynthetic process, while GO-cellular component (CC) analysis showed that candidate genes were mainly located in endomembrane system and organelle membrane, with molecular functions (MF) mainly including aromatase activity, metal ion binding, and cation binding (**Figure 5C**).

To discover the potential prognostic significance of key genes of AA metabolism, we performed a univariate Cox hazard regression analysis. The expressions of 3 genes were found to be significantly associated with OC patient with overall survival(OS) and 2 genes associated with OC patient with progression-free survival (PFS) (**Figures 5D, E**).

## 7 Prospect

With the continuous development of research methods and tools, it has been gradually discovered that AA and its metabolites play important roles in the proliferation, metastasis, apoptosis, angiogenesis, and inflammatory responses of various tumor cells such as ovarian cancer, as well as in treatment and prognosis. High levels of COX, LOX, CYP, and key enzymes in related metabolic pathways may serve as potential targets for early detection and treatment of ovarian cancer. These pathways have been utilized to assess the progression of ovarian cancer, which is crucial for clinical diagnosis of the disease, especially early diagnosis. As an important metabolite of AA metabolism, an increased level of 15(S)-HETE has been identified in various cancers, including non-small cell lung cancer and breast cancer, which suggests that 15(S)-HETE could be served as a potential biomarker for cancer diagnosis (44).245 epithelial ovarian cancer samples were explored by tissue microarray and revealed a higher expression of 12-LOX. Furthermore, it was found that free fatty acid metabolism via LOX pathway leads to an elevated level of 8-HETE in women at risk for developing ovarian cancer. Therefore, measuring levels of 8-HETE could be proposed as an important indicator for assessing the risk of ovarian cancer (91).

This review emphasizes the critical role of AA and its metabolites in the occurrence, development, and metastasis of ovarian cancer. However, due to the complex biochemical nature of AA and its metabolites, their role in ovarian cancer remains challenging. Further research into the relationship between AA and tumor development is essential, requiring a more comprehensive and systematic exploration of the association between inhibitors and combination therapy efficacy. It is anticipated that with the discovery of more biomarkers, AA will usher in a new era of gene-targeted cancer therapy.

## Author contributions

QX: Visualization, Writing – original draft. WG: Conceptualization, Writing – review & editing. JY: Formal Analysis, Writing – review & editing. ZX: Project administration, Writing – review & editing. ZJ: Funding acquisition, Writing – review & editing.
