# Supplementary material for: The deregulation of arachidonic acid metabolism in ovarian cancer
Source: Front Oncol. 2024 May 2;14:1381894. doi: 10.3389/fonc.2024.1381894 (PMC11100328; doi:10.3389/fonc.2024.1381894)

	pvalue	Hazard ratio
EGFR	0.002	1.069(1.025−1.115)
PTGIS	0.003	1.014(1.005−1.022)
ALOX5AP	0.028	1.006(1.001−1.011)
HPGD	0.076	0.989(0.977−1.001)
ALOX5	0.113	1.015(0.996−1.034)
ALOX15	0.152	0.845(0.672−1.064)
IL1B	0.153	1.027(0.990−1.064)
PTGES	0.202	1.010(0.994−1.027)
GSTP1	0.210	1.000(0.999−1.000)
CYP1A1	0.210	1.160(0.920−1.462)
NOS2	0.212	1.620(0.760−3.455)
CYP2A13	0.226	1.152(0.916−1.448)
CYP1A2	0.291	1.035(0.971−1.103)
ALOXE3	0.322	0.881(0.685−1.132)
TNF	0.377	0.992(0.975−1.010)
COX5A	0.396	0.999(0.997−1.001)
GSTM1	0.398	0.995(0.985−1.006)
CYP3A4	0.422	0.434(0.057−2.322)
PTGS1	0.425	1.001(0.998−1.004)
PPARG	0.444	1.043(0.936−1.162)
PTGER4	0.501	1.025(0.953−1.103)
LTC4S	0.527	1.003(0.994−1.013)
COTL1	0.579	1.005(0.988−1.021)
ALOX12B	0.716	0.937(0.660−1.330)
GPX4	0.723	1.000(0.998−1.001)
CYSLTR1	0.772	1.013(0.928−1.106)
ABCB1	0.868	0.979(0.761−1.258)
CYP2E1	0.891	1.036(0.626−1.715)
NR1I2	0.927	0.977(0.594−1.607)

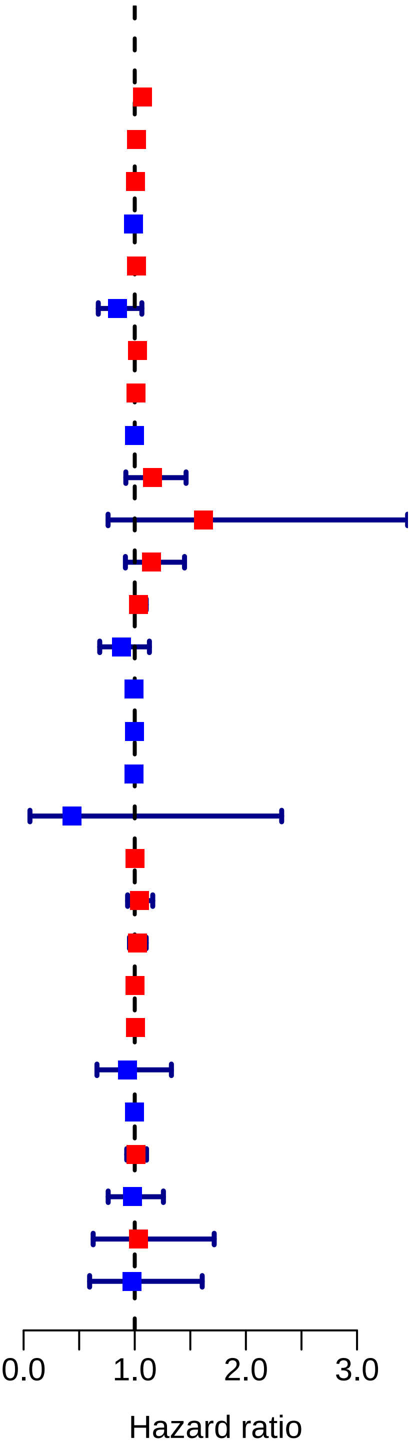

Supplement: Supplementary file 1 [file DataSheet_1.zip › Supplementary Material/OS and PFS/os/38.forest/forest.pdf]
