# Supplementary material for: The deregulation of arachidonic acid metabolism in ovarian cancer
Source: Front Oncol. 2024 May 2;14:1381894. doi: 10.3389/fonc.2024.1381894 (PMC11100328; doi:10.3389/fonc.2024.1381894)

	pvalue	Hazard ratio
PTGIS	0.031	1.010(1.001−1.019)
CYP2A13	0.034	1.244(1.017−1.521)
PTGER4	0.057	0.929(0.861−1.002)
EGFR	0.078	1.039(0.996−1.083)
PTGES	0.130	0.989(0.974−1.003)
TNF	0.178	0.989(0.973−1.005)
PTGS1	0.236	0.998(0.996−1.001)
GSTP1	0.330	1.000(1.000−1.001)
ALOX5AP	0.369	1.003(0.997−1.009)
ALOX5	0.386	1.008(0.991−1.025)
HPGD	0.388	0.996(0.987−1.005)
GSTM1	0.390	0.995(0.985−1.006)
CYSLTR1	0.455	1.030(0.954−1.112)
NR1I2	0.496	0.857(0.551−1.336)
GPX4	0.517	1.000(0.998−1.001)
ALOX15	0.522	0.935(0.760−1.150)
CYP1A1	0.573	0.925(0.705−1.213)
CYP2E1	0.618	0.888(0.558−1.415)
CYP3A4	0.632	0.622(0.090−1.322)
LTC4S	0.656	1.002(0.994−1.010)
ALOXE3	0.667	0.961(0.803−1.151)
ALOX12B	0.737	1.057(0.764−1.464)
IL1B	0.817	1.004(0.970−1.039)
CYP1A2	0.831	1.008(0.934−1.089)
PPARG	0.871	1.008(0.911−1.116)
ABCB1	0.874	1.022(0.786−1.328)
COTL1	0.929	1.001(0.986−1.016)
NOS2	0.942	1.029(0.473−2.241)
COX5A	0.952	1.000(0.998−1.002)

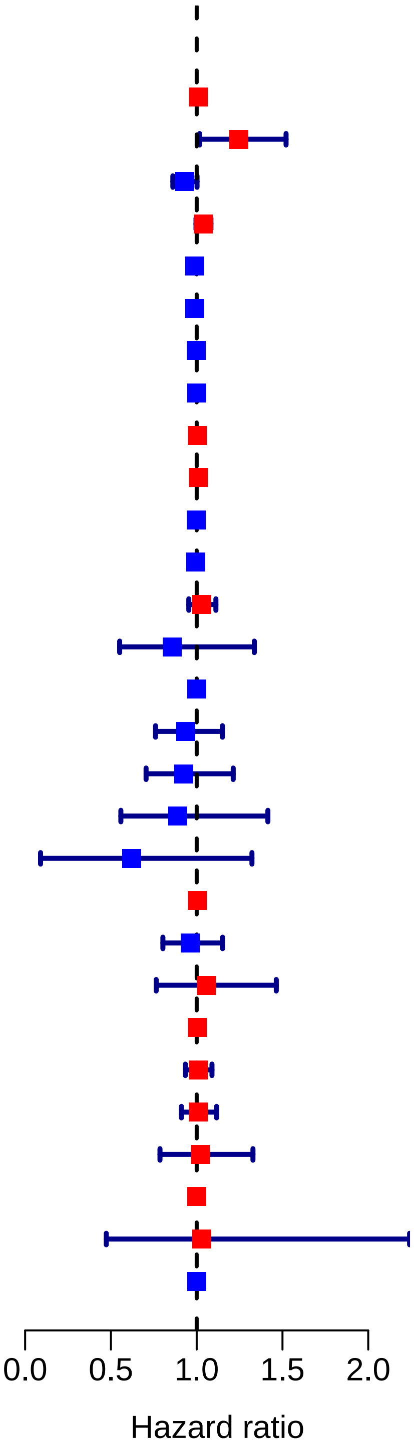

Supplement: Supplementary file 1 [file DataSheet_1.zip › Supplementary Material/OS and PFS/PFS/38.forest/forest.pdf]
